# Clinical and Financial Impact of Rapid Antimicrobial Susceptibility Testing in Blood Cultures

**DOI:** 10.3390/antibiotics11020122

**Published:** 2022-01-18

**Authors:** Felix Roth, Nathan D. Leedahl, David D. Leedahl, Dubert M. Guerrero

**Affiliations:** 1 Sanford Health, Fargo, ND 58104, USA; Felix.Roth@sanfordhealth.org (F.R.); Nathan.Leedahl@sanfordhealth.org (N.D.L.); david.leedahl@gmail.com (D.D.L.); 2 School of Medicine and Health Sciences, University of North Dakota, Fargo, ND 58102, USA

**Keywords:** antimicrobial susceptibility testing, blood culture, rapid identification

## Abstract

The rapid identification of pathogens that cause bloodstream infections plays a vital role in the modern clinical microbiology laboratory. Despite demonstrating a significant reduction in turnaround time and a significant effect on clinical decisions, most methods do not provide complete antimicrobial susceptibility testing (AST) information. We employed rapid identification (ID) and AST using the Accelerate PhenoTest on positive blood cultures containing Gram-negative bacilli. The length of stay (LOS) significantly decreased from an average of 12.1 days prior to implementation to 6.6 days post-implementation (*p* = 0.02), representing potential total savings of USD 666,208.00. All-cause mortality did not differ significantly, 27 (19%) versus 18 (12%), *p* = 0.11. We also observed an associated decrease in the use of broad-spectrum antimicrobials, including meropenem and quinolones. The implementation of a rapid ID and AST method, along with a well-established antimicrobial stewardship program, has the potential to decrease LOS, broad-spectrum antibiotic use, and costs to the healthcare system, with no observable impact on mortality.

## 1. Introduction

Bloodstream infections (BSI) remain a significant cause of morbidity and death, most notable among the elderly and other high-risk populations, with an approximate mortality of up to 80% [1,2,3]. Prompt initiation of effective antibiotics, identification of the etiologic cause, and antimicrobial susceptibility testing (AST) have the potential to reduce this incidence significantly and, therefore, improve patient care [3,4]. The utilization of rapid molecular methods in the form of microarrays to identify the potential cause of BSI has irrefutably improved the management of BSI [5,6,7]. However, most of these platforms are unable to provide information on susceptibilities and still rely on semi-automated methods that require at least 18 h of incubation, which may lead to potential delays in accessing appropriate antimicrobials. Additionally, the emergence of drug resistance poses additional challenges and emphasizes the need for the early identification of AST results [8]. A new FDA-approved platform, Accelerate Pheno^TM^ (ACC) (Accelerate Diagnostics, Tucson, AZ), offers a solution for rapid AST in positive blood culture specimens [9,10]. To our knowledge, this is the only United States Food and Drug Administration (FDA)-cleared platform using a morphokinetic approach to obtain AST results; its implementation posed a reliable solution to expedite AST results and improve patient care. Adopting the ACC test reduced the turnaround time, with results being reported within seven hours, compared to traditional methods, which can take as long as 48 h to report results. This study reports the economic and clinical impact of adopting ACC in a teaching community hospital.

## 2. Results

There were 142 patients in the pre-ACC and 148 patients in the post-ACC implementation periods (Table 1). A number of the blood cultures were polymicrobial, and the most common organism isolated was E. coli for both the pre-ACC and post-ACC patients, in 120 (52%) and 160 (59%) patients, respectively. This was followed by *Klebsiella pneumoniae* and *Pseudomonas aeruginosa*. There were no statistical differences in the types of organisms isolated in both groups. All-cause mortality during or one month after admission occurred in 27 (19%) patients in the pre-ACC period versus 18 (12%) patients in the post-ACC period, and was not significant (*p* = 0.11). The length of stay at the hospital significantly decreased from 12.1 ± 2.0 days in the pre-ACC patients to 6.6 ± 8.3 days in the post-ACC group (*p* = 0.02). The *C. difficile* rates were similar in the pre-ACC period, with a median of 5.86/10,000 patient days, and the post-ACC period, with a median of 4.94/10,000 patient days (*p* = 0.71). Overall, antibiotic use increased from 598.9 days of therapy (DOT)/1000 patient days pre-implementation to 621.8 DOT/1000 patient days post-implementation. However, broad-spectrum antibiotic use, specifically of carbapenems, decreased from 18.9 DOT/1000 patient days to 14.6 DOT/1000 patient days, and quinolones from 25.3 DOT/1000 patient days to 19.8 DOT/1000 patient days. Meropenem is our institution’s carbapenem in the formulary. The total days of meropenem therapy decreased from 249 in the pre-group to 115 days in the post-group. This was a potential antibiotic annual cost saving of USD 8,169, but the difference of 5 days in length of stay has a greater potential saving. In all the facilities in our institution, we projected 468 encounters a year for Gram-negative BSI. This translates to 4,685 total days in the hospital attributable to Gram-negative BSI, based on an average LOS of 10.01 days, resulting in a reduction of 1546 days. With an average cost of USD 550 per day to our healthcare system, we project an annual cost saving of USD 850,328. The annual cost to perform the test is USD 184,120, resulting in an annual net saving of USD 666,208.

## 3. Discussion

In this study, we report real institutional experience in implementing rapid detection and antimicrobial susceptibility testing using the ACC platform on Gram-negative bloodstream infections. The main impact was an overall decrease in hospital stay from 12.1 to 6.6 days, which translated to a projected cost saving of over USD 660,000 a year. Gram-negative antimicrobial use, specifically of quinolones and meropenem, has decreased, with no observable effect on overall mortality or the emergence of nosocomial infections, specifically *C. difficile* infection.

ACC is a fully automated test system that is able to identify a pathogen and conduct susceptibility testing directly from positive blood cultures. It is a rapid test, with results available within approximately seven hours. The system uses a combination of gel electrofiltration and fluorescence in situ hybridization for identification, and automated microscopy for bacterial growth rate analysis, which is used for extrapolating the minimum inhibitory concentration (MIC) values. The use of this FDA-approved platform, in conjunction with a well-established ASP, can potentially impact patient care and management [9,10].

A benefit of combining rapid identification and antimicrobial susceptibility testing is the potential to move patients’ antibiotics from empiric to optimal therapy faster, using the results of MIC. In a recent study using ACC, it was determined that almost half of patients with resistant Gram-negative infections had potential for improvement in time to effective therapy (TTET) and time to definitive therapy (TTDT) of almost 17 h and 30 h, respectively [11]. A meta-analysis review demonstrated decreased TTET, LOS and mortality risk of bloodstream infections if molecular rapid testing is utilized in conjunction with an effective antimicrobial stewardship program, compared to conventional microbiologic methods [12,13]. This strategy and the utilization of molecular diagnostic tests are supported by the Infectious Disease Society of America (IDSA), and address the issue of expediting the integration of improved diagnostic tests into patient care, as outlined in an IDSA policy paper [14,15].

Other than outcomes such as decreased mortality risk and LOS, the use of rapid diagnostic tests, such as ACC, has a potential impact on healthcare cost [16,17,18,19]. A mean LOS of 10 days and cost of USD 43,208.00 were found to be attributable to BSI in a study across 8 hospitals in South-Central United States [1]. We projected a direct net cost saving of over USD 660,000 a year, due to a reduction in LOS and reduced acquisition cost of broad-spectrum antibiotics, such as carbapenems. This is an underestimation due to other potential attributable costs, such as nursing staff, ICU stays, laboratory testing, diagnostic imaging, potential surgery, pharmacy, and other support services associated with longer hospital stays.

In conclusion, the implementation of a rapid identification (ID) and AST method in our institution, along with a well-established antimicrobial stewardship program, significantly decreased LOS, broad-spectrum antibiotic use, and costs to the healthcare system, with no observable impact on mortality.

## 4. Materials and Methods

We implemented ACC for rapid ID and AST at Sanford Medical Center Fargo on positive blood cultures containing Gram-negative bacilli in 2019. Prior to that, the Verigene BC test (Nanosphere, Northbrook, IL, USA) for rapid ID on positive blood cultures was employed. The Verigene BC test already informs providers of the cause of a bloodstream infection 24–48 h earlier than conventional identification methods [20]. However, it does not provide information regarding specific antimicrobial susceptibilities, a critical component to achieving adequate treatment, which is especially challenging in this era of antimicrobial resistance. The Sanford Health IRB authorized this study (STUDY00001159) under an HIPAA Waiver or Alteration of Authorization.

Seven months of post-implementation data were obtained, which included length of hospital stay, all-cause mortality one month after hospital admission, antimicrobial use, *Clostridium difficile* infection and predicted direct healthcare costs. Patient charts were accessed in order to retrieve and analyze laboratory results, duration of antimicrobial therapy and length of stay. The data analytics team in our institution developed specific reports to collect the information presented in this submission; they also verified accuracy for length of stay calculations and costs. A retrospective comparison study was conducted comparing the parameters prior to implementing ACC within an equivalent 7 month period with the previous year (pre-group). All patients with a Gram-negative microorganism isolated from a blood culture during hospitalization were included in the analysis. Antimicrobial stewardship program (ASP) pharmacists were actively involved in both phases of the study, facilitating timely modifications to antimicrobial therapy. No other changes in ASP protocols or formulations were introduced during the study period. Data were analyzed using a Student’s t-test for continuous variables and chi-square test for categorical variables. All the statistical tests were two tailed with *p* < 0.05 considered to be significant.

## Figures and Tables

**Table 1:** Patient characteristics, culture information and outcome information before and after implementation of rapid identification and antimicrobial susceptibility testing using a new FDA-approved platform, Accelerate Pheno^TM^ (ACC) (Accelerate Diagnostics, Tucson, AZ, USA).

Patients’ Demographic Characteristics, Bacterial Isolates and Outcome in Pre- and Post-ACC Implementation Period			
	Pre-ACC (*n* = 142)	Post-ACC (*n* = 148)	*p*-Value
Age, median [range]	67 [3–92]	65 [0–91]	0.55
Male (%)	72 (51)	67 (45)	0.41
Organism	229	291	
*Escherichia coli*	120 (52)	160 (59)	0.56
*Klebsiella pneumoniae*	33 (14)	37 (14)	0.57
*Pseudomonas aeruginosa*	35 (15)	26 (10)	0.03
*Enterobacter* spp.	19 (8)	15 (6)	0.15
Other	22 (10)	35 (13)	0.38
Mortality	27 (19)	18 (12)	0.11
Length of stay, days	12.1 ± 2.0	6.6 ± 8.3	0.02
*Clostridium difficile*/10,000 patient days	5.86	4.94	0.71

## Data Availability

The data presented in this study are available on request from the corresponding author.
